# Effect of Dietary Supplementation with Lutein, Zeaxanthin, and Elderberries on Dry Eye Disease (DED) and Immunity: A Randomized Controlled Trial

**DOI:** 10.3390/nu16244366

**Published:** 2024-12-18

**Authors:** Kok Ming Goh, Eugenie Sin Sing Tan, Crystale Siew Ying Lim, Pui Yee Tan, Sayantan Biswas, Li Ann Lew, Chung Keat Tan

**Affiliations:** 1 New Product Development Department, Ecolite Biotech Manufacturing, Yong Peng 83400, Malaysia; gohkm@ecolite.com.my; 2 Product Development Department, Xmegami Manufacturing, Puchong 47170, Malaysia; la.lew@xmegamimfg.com; 3 Faculty of Medicine and Health Sciences, UCSI University, Kuala Lumpur 56000, Malaysia; eugenietan@ucsiuniversity.edu.my; 4 Faculty of Applied Sciences, UCSI University, Kuala Lumpur 56000, Malaysia; crystalelim@ucsiuniversity.edu.my; 5 Faculty of Environment, University of Leeds, Leeds LS2 9JT, UK; prcpyt@leeds.ac.uk; 6 School of Optometry, College of Health and Life Sciences, Aston University, Birmingham B4 7ET, UK; s.biswas2@aston.ac.uk; 7 Faculty of Food Science and Technology, Universiti Putra Malaysia, Serdang 43400, Malaysia

**Keywords:** lutein, zeaxanthin, elderberries, Ocular Surface Disease Index (OSDI), Immune Status Questionnaire (ISQ)

## Abstract

Background/Objectives: Dry eye disease (DED) significantly impairs quality of life, affecting physical, social, and psychological well-being, as well as reducing workplace productivity. While lutein and zeaxanthin supplements have been shown to improve ocular health, existing research often overlooks the efficacy of lower dosages and shorter durations of supplementation. This study investigated the effects of combined supplementation with lutein, zeaxanthin, and elderberries in 110 voluntary participants through a randomized controlled trial. Methods: Participants took 6 mg of lutein and 1 mg of zeaxanthin, along with 100 mg elderberry extract once daily for a duration of 20 days. Ocular health was assessed using the Ocular Surface Disease Index (OSDI), while immune status was evaluated with the Immune Status Questionnaire (ISQ). Results: Results showed that combined supplementation significantly (*p* < 0.05) reduced the OSDI scores in the intervention group from 38.15 ± 11.14 to 18.26 ± 5.57, reflecting a 52.2% reduction. A similar trend was observed with the Visual Analog Scale (VAS), indicating significant (*p* < 0.05) improvement from 5.31 ± 1.62 to 6.73 ± 1.74, equivalent to a 26.7% improvement. Although the intervention group showed a 15.9% improvement in ISQ scores by the study’s end, this was not significantly different from the placebo group, suggesting that higher dosages or longer durations may be needed to observe a meaningful effect. Additionally, findings from the Food Frequency Questionnaire revealed that the average dietary intake of lutein and zeaxanthin among participants was only 663.49 µg, equating to just 5.5% of the suggested optimal daily intake. This low consumption is concerning, as it is inversely correlated with the risk of ocular diseases. Conclusions: Collectively, these findings support the use of combined supplementation as an adjuvant approach to improving ocular health.

## 1. Introduction

Dry eye disease (DED) is a complex, multifactorial disease that affects the tear film and ocular surface, resulting in symptoms such as itching, grittiness, a foreign body sensation, excessive tearing, visual disturbances, and tear film instability [1]. This condition may also damage the ocular surface, accompanied by increased tear film osmolarity and ocular surface inflammation [2]. Notable risk factors for DED include advancing age, East and South Asian ethnicity, contact lens use, prolonged digital screen exposure, elevated psychological stress, and environmental factors such as low temperature and humidity [3,4]. DED significantly impairs quality of life, affecting physical, social, psychological well-being, as well as reducing workplace productivity [5]. The global prevalence of DED ranges from 5% to 50%, depending on the geographic region [6]. Advanced age, female sex, a history of postmenopausal estrogen therapy, prior ocular surface surgery, and the use of antihistamine medications have been identified as primary risk factors [7,8]. Additional risk factors include environmental influences such as extreme temperatures, air pollution, low relative humidity, prolonged use of visual display terminals (VDUs), smoking and contact lens wear [9,10]. Furthermore, DED is often associated with comorbidities such as diabetes mellitus, rosacea, hypertension, thyroid disease, gout, arthritis, osteoporosis, and other systemic conditions [8,11].

The potential role of nutritional interventions in promoting ocular health has been extensively studied over several decades [12,13]. Lutein and zeaxanthin are well-recognized for their antioxidant properties and capacity to filter high-energy blue light produced by visual display units (VDU) [14,15]. Lutein and zeaxanthin are xanthophyll carotenoids found in carrots, corn, grapes, kiwi, orange peppers, spinach and zucchini; commonly found in green leafy vegetables as well as orange and yellow fruits and vegetables [16,17]. These carotenoids are particularly concentrated in the macula lutea, the central part of the human retina responsible for high-resolution vision [18]. Although a daily intake of 10 mg of lutein and 2 mg of zeaxanthin is recommended to support ocular health, actual consumption levels within the general population are often significantly lower than these dietary targets [19]. For instance, the average daily intake is approximately 2.7 mg for men and 3.09 mg for women among older American adults, while older Australian adults consume even less, averaging around 0.9 mg per day [20,21]. This discrepancy highlights a significant gap between recommended and actual dietary intake, which may affect eye health in these populations.

Elderberries have been recognized for millennia for their nutritional and therapeutic benefits. They possess various bioactive properties, including immune-stimulating, and antiviral, antioxidant, and antibacterial effects [22,23,24,25]. Moreover, elderberries show potential in antitumor activity, managing obesity and metabolic disorders, providing antidepressant effects and exhibiting antidiabetic properties [26,27,28,29]. These medicinal properties are largely attributed to their high content of polyphenols, including anthocyanins, flavanols, and proanthocyanins, which contribute to their high antioxidant and anti-inflammatory capacities [30]. Anthocyanins are believed to exert their antiviral effects by disrupting the entry virus to the host cells and suppressing the inflammation levels by inhibiting MAPK signaling pathways [31,32]. Although elderberries’ bioactive properties have been well-documented in animal and in vitro studies, the paucity of comprehensive clinical trials limits the confirmation of these benefits in humans, underscoring the necessity for rigorous research [30,33].

Effective management of dry eye disease (DED) often requires a multifaceted therapeutic approach [34]. Most ophthalmic treatments involve topical eyedrops, but their therapeutic effectiveness is frequently hindered by rapid clearance from the precorneal space due to blinking, nasolacrimal drainage, and both reflex and basal tear production [35]. These limitations have prompted research into antioxidant-rich nutraceuticals as safer and potentially more effective alternatives to conventional ophthalmic drugs, aiming to improve patient outcomes and adherence [36]. High-dose supplementation with lutein and zeaxanthin, at levels exceeding 10 mg/day, has been shown to enhance ocular health by increasing macular pigment optical density (MPOD) [18,37,38]. However, studies using low doses of lutein and zeaxanthin have produced inconclusive results, often due to insufficient data, lack of randomization, and absence of blinding [39]. This highlights the need for future research to establish the minimum effective dose and duration for lutein and zeaxanthin supplementation necessary to achieve clinically significant improvements in ocular health. Therefore, this study aims to evaluate the effects of combined supplementation with lutein, zeaxanthin, and elderberries on enhancing ocular health and boosting immune responses.

## 2. Materials and Methods

### 2.1. Participant Recruitment

**Figure 1:**
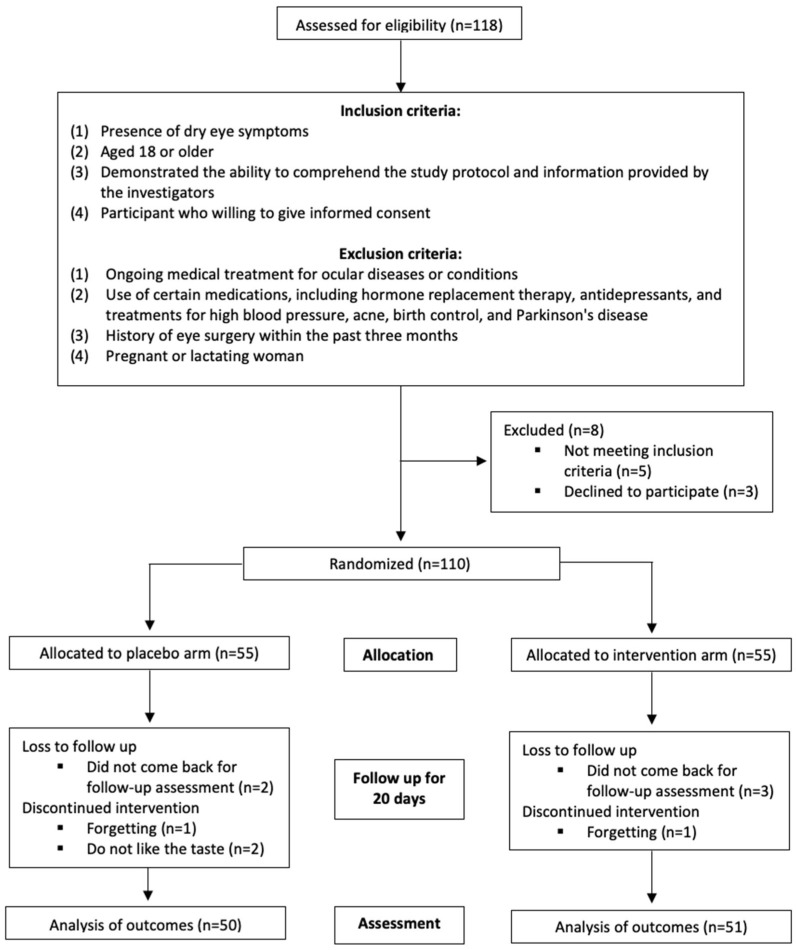
CONSORT protocol for the study described with flow diagram.

This randomized, double-blind, placebo-controlled study involved a twenty-day supplementation period and included 110 participants. The inclusion criteria were as follows: (1) presence of dry eye symptoms, (2) age 18 years or older, (3) ability to comprehend the study protocol and information provided by the investigators, and (4) provision of informed consent. Exclusion criteria encompassed the following: (1) ongoing medical treatment for ocular diseases or conditions; (2) use of certain medications, including hormone replacement therapy, antidepressants, and treatments for high blood pressure, acne, birth control, and Parkinson’s disease; (3) history of eye surgery within the past three months; and (4) pregnancy or lactation (Figure 1). This study was registered in Clinicaltrials.gov with the ID NCT06574685.

The participants received detailed information through a participant information sheet and a thorough explanation from the investigators. Written informed consent was obtained from each participant. Randomization into the control or intervention group was performed using a dice-rolling system. To maintain blinding, the placebo supplements were identical in appearance to the intervention supplements, and the investigators remained unaware of the participants’ group assignments. Participant compliance was monitored through a compliance form. This study adhered to the ethical principles outlined in the Declaration of Helsinki and the Malaysian Guidelines for Good Clinical Practice. Ethical approval was granted by the UCSI Institutional Ethics Committee (IEC) under the approval code UCSI-IEC-2024-FMHS-0003 (A).

### 2.2. Supplementation

The intervention supplement consisted of marigold extract, zeaxanthin, elderberry extract, bilberry extract, lycopene extract, multivitamin, and inulin, all in powder form and packaged in individual sachets (Twinkle Eyez™; Xmegami, Puchong, Selangor, Malaysia). Each sachet contained 6 mg of lutein and 1 mg of zeaxanthin, along with 100 mg of elderberry extract. The active ingredients were extracted using the conventional water-based decoction method at 100 °C. It is important to note that the chemical composition and forms of the ingredients may undergo changes during the extraction process. However, investigating these changes is beyond the scope of this study. The placebo supplement was identical in composition but excluded lutein, zeaxanthin, and elderberry extract. The participants were instructed to prepare the beverages by mixing the contents of each sachet with 150 mL of lukewarm water and to consume them once daily before meals. All the supplements were labeled in a blinded manner, and the participants were advised to maintain their usual dietary and physical activity habits throughout the 20-day supplementation period.

### 2.3. Instruments (Participant-Reported Outcomes)

At the baseline visit, demographic and anthropometric data were collected from the participants. Dietary intakes of lutein and zeaxanthin over the previous six months were assessed using a validated, simplified food frequency questionnaire (FFQ) tailored to include 30 food items known for their lutein and zeaxanthin content [17]. For each food item, a specific serving size was provided, and the participants reported their consumption frequency in terms of ‘never’ or the number of occasions per day, week, or month, as appropriate. The dietary intake of lutein and zeaxanthin was then calculated based on values from the United States Department of Agriculture–National Cancer Institute Carotenoids Database [40].

Three instruments were administered at both the baseline and post-treatment visits, which occurred after 20 days: the Ocular Surface Disease Index (OSDI), the Immune Status Questionnaire (ISQ), and the Visual Analog Scale (VAS). The OSDI, a validated 12-item questionnaire, assesses three subcategories: ocular symptoms, vision-related function, and environmental triggers. Each item is rated on a scale from 0 (‘none of the time’) to 4 (‘all of the time’), with the final OSDI score calculated by summing the participant’s responses [41]. ISQ is a validated questionnaire commonly used in both research and clinical settings [42]. The ISQ consists of seven items that assess the occurrence of various immune-related symptoms in the past. The total ISQ score was recorded onto a scale from 0 (very poor) to 10 (excellent). The VAS is a psychometric measuring instrument designed to document the characteristics of disease-related symptom severity or general well-being of an individual, often used in epidemiological and clinical research [43,44,45]. The participants were asked to self-rate their current overall ocular health and immunity status using a 10 cm VAS, where 0 indicated a very poor condition and 10 indicated an excellent condition.

### 2.4. Statistical Analysis

The participant characteristics were presented as categorical data, expressed in frequencies and percentages. All the outcomes were analyzed as continuous dependent variables and reported as mean ± SD. Changes in the OSDI, ISQ and VAS scores were analyzed using a general linear model (GLM) for repeated measures. The within-subjects variable was defined as the sampling time point, while the grouping was tested as a between-subject factor. The homogeneity of the variance and covariance structure of the dependent variables was assessed using Box’s M and Levene’s tests. The sphericity of residual covariance matrix was evaluated using Mauchly’s sphericity test. The results were considered significant if *p* < 0.05, with a 95% confidence interval. Statistical analysis was performed using SPSS version 29.0 (IBM Corp., New York, NY, USA) for MacOS.

## 3. Results

### 3.1. Characteristics of Participants

A total of 110 participants who reported dry eye symptoms within the past three months were recruited for the study. Of these, four participants were excluded due to low compliance, and five were lost to follow-up, resulting in a drop-out rate of 8.2% (Figure 1). The majority of the participants were aged between 41 and 50 years (*n* = 48, 47.6%), followed by those aged 31 to 40 years (*n* = 21, 20.8%), those older than 50 years (*n* = 18, 17.8%), and those aged 30 years or younger (*n* = 14, 13.8%). The female participants (*n* = 62, 61.4%) outnumbered the male participants (*n* = 39, 38.6%). Most of the participants were of normal weight (*n* = 51, 50.5%), followed by those classified as overweight (*n* = 35, 34.7%). The proportions of participants who were underweight or obese were 6.9% and 7.9%, respectively (Table 1).

### 3.2. Daily Intakes of Lutein and Zeaxanthin

The average daily total intake of lutein and zeaxanthin among the study participants was 663.49 µg. The highest contributions to this intake were from cooked kale (140.77 µg), cooked spinach (110.21 µg), and raw romaine lettuce (74.54 µg). These findings highlight the significant role of dark green leafy vegetables as major sources of lutein and zeaxanthin in the diet. In contrast, the lowest intakes were observed from fruits such as melon (0.41 µg), peach (0.21 µg), and grapefruit (0.07 µg). The markedly lower intake from these fruits underscores the relatively limited contribution of fruit sources to overall lutein and zeaxanthin consumption compared to vegetables (Table 2).

### 3.3. Changes in Ocular Health Parameters

Our results indicate a significant reduction in Ocular Surface Disease Index (OSDI) scores within the intervention group, decreasing from 38.15 ± 11.14 to 18.26 ± 5.57 post-treatment, reflecting a 52.2% reduction. This decrease was significantly greater (*p* < 0.05) than the reduction observed in the placebo group. Additionally, the findings demonstrated that supplementation significantly improved the Visual Analog Scale (VAS) scores in the intervention group, increasing from 5.31 ± 1.62 to 6.73 ± 1.74, a 26.7% improvement, which was significantly better (*p* < 0.05) compared to the effects observed in the placebo group (Table 3). The results did not show any significant differences when gender, age, and BMI were being tested as between-subject effects.

### 3.4. Changes in Immunity Parameters

The changes in immunity among the participants were evaluated using the Immune Status Questionnaire (ISQ) and the Visual Analog Scale (VAS). In the intervention group, the ISQ scores improved from 7.92 ± 2.45 to 9.18 ± 1.91 post-treatment, representing a 15.9% increase attributed to the supplementation. However, this improvement was not significantly different from the 10.0% improvement observed in the placebo group. Similarly, the VAS scores showed a comparable trend to the ISQ scores, increasing from 6.53 ± 1.59 to 6.82 ± 1.88 post-treatment, but the changes were not statistically different from those observed in the placebo group (Table 4). The results did not show any significant differences when gender, age, and BMI were being tested as between-subject effects.

## 4. Discussion

Dry eye disease (DED) is a highly prevalent ocular surface condition worldwide, increasingly recognized as a significant public health concern due to its rising prevalence in recent years [46]. Key risk factors for dry eye disease include aging, ethnicity, contact lens use, extended digital screen time, increased psychological stress, and environmental conditions like low temperature and humidity [3,4]. A comprehensive Bayesian analysis of the data collected between 1997 and 2021 estimates the global prevalence of DED at 29.5%, with notable regional disparities: North America exhibits the lowest prevalence at 3.5%, while Eastern Asia reports the highest at 42.8% [47]. In a previous study conducted in a specific state in Malaysia, the crude prevalence of DED was 48.5%, with an age-adjusted prevalence of 68.4% among women over 50 years old [48]. This high morbidity leads to a loss of quality of life and represents a substantial economic and humanistic burden on national healthcare systems [49]. The wide range of prevalence underscores the complex interplay of lifestyle, dietary, genetic, and environmental factors in the pathophysiology of DED [7,8,9]. Gender and age stand out as two of the most significant risk factors for DED. Consistent with our study’s findings, research has repeatedly shown that DED is more prevalent in women than in men. This higher prevalence in women is likely driven by lower androgen levels and exogenous estrogen use [11,50,51]. Furthermore, a recent large cohort study revealed that women are diagnosed with DED, on average, six years earlier than men [52]. Age also emerges as a critical risk factor, with numerous studies demonstrating an increase in DED incidence with advancing age, corroborating our findings of higher prevalence among participants over 40 years old [3,53,54]. This age-specific prevalence may be attributed to age-related changes in physiological functions, hormones, lifestyle, and diet [54,55,56,57]. Overall, these findings highlight the importance of considering gender and age in the development of DED management plans, as early diagnosis and intervention could significantly enhance patients’ quality of life.

Two critical tissues involved in the visual process are the macula and the lens. Among the carotenoids present in the human body, lutein and zeaxanthin are the only ones found in these tissues. Zeaxanthin predominantly accumulates in the central macula, while lutein is more concentrated in the peripheral parts [18]. Given their accumulation in the retina, lutein and zeaxanthin are known for their protective roles against the most harmful blue visible light (400–500 nm) from the environment [14]. Additionally, their antioxidant properties help protect the macula from photochemical damage [15]. Lutein and zeaxanthin are essential nutrients that can only be obtained from diet. Sources of lutein and zeaxanthin include green leafy vegetables, corn, zucchini, broccoli, and carrot [16,17]. Although substantial evidence suggests that lutein and zeaxanthin play a protective role against certain chronic diseases, there are no defined Recommended Dietary Allowance (RDA) or Recommended Daily Intake (RDI) for these carotenoids currently. Strong evidence suggests that an optimal daily intake of 10 mg of lutein and 2 mg of zeaxanthin is important for maintaining healthy visual function [19,58,59,60]. However, the actual intake of these carotenoids is generally low, with reported daily intakes of approximately 1–2 mg among American adults [61], even lower in developing countries, such as Indonesia (1.03 mg) [62], Cuba (0.91 mg) [63], and Brazil (0.86 mg) [64]. These findings align with the current study, which reported an average daily intake of 0.663 mg among participants, equating to only 5.5% of the suggested optimal daily intake. This is concerning, as low consumption of lutein and zeaxanthin has been shown to be inversely correlated with the risk of age-related macular degeneration (AMD), a leading cause of blindness [65,66], as well as other ocular diseases [67,68,69,70,71]. This concern is underscored by data from the 2023 National Health and Morbidity Survey (NHMS), which reported that 11% of the Malaysian population experiences some degree of visual impairment [72]. Similarly, a global meta-analysis also highlighted that the majority of visually impaired individuals reside in South Asia, East Asia, and Southeast Asia [47].

As observed in our study, a substantial part of the population fails to meet current vitamin recommendations through diet alone. As a result, dietary supplements have emerged as a practical solution to fulfill daily nutritional needs and serve as a preventive measure against various diseases [73,74,75]. The use of lutein and zeaxanthin supplements, particularly for ocular health, has gained popularity since their introduction in the late 1990s [76]. According to recent National Health and Nutrition Examination Survey (NHANES) data, the prevalence of lutein and zeaxanthin supplement use is approximately 4% among adults aged 20 and older, with this figure doubling in individuals over 60 years of age [77]. High-dose lutein and zeaxanthin supplementation has been proven effective in increasing macular pigment optical density (MPOD) [18,37,38]. However, the effects of low doses of lutein and zeaxanthin remain underexplored, as most studies have focused on dietary interventions, complicating the distinction between dosage effects and dietary sources [39]. Notably, the two trials that examined supplement interventions at lower doses reported contradictory outcomes [78,79]. Additionally, the majority of these trials spanned 3 to 24 months, making them susceptible to confounding factors such as changes in dietary intake and adherence [39]. This underscores the need to determine the minimum supplementation duration required to achieve clinically significant improvements in visual function. The Ocular Surface Disease Index (OSDI) is a widely used 12-item questionnaire that rapidly assesses ocular irritation symptoms associated with DED and their impact on vision-related functioning [41]. OSDI is the most widely used patient reported outcomes for dry eyes symptoms in clinical studies, with a documented application in over 600 records [80]. Our results show that a 20-day combined supplementation regimen significantly reduced OSDI scores by 52.2%, a substantial improvement compared to the placebo group. This aligns with previous research indicating that a 30 mg daily dose of zeaxanthin can achieve a plateau in MPOD within 30 days [79]. This observation suggests that the combined supplementation used in this study can produce effects comparable to high-dose zeaxanthin supplementation. The improvement in dry eye symptoms may be attributed to the beneficial effects of lutein and zeaxanthin, which likely enhance tear production, stability, and quality, while also reducing ocular surface inflammation [81]. The improvement in DED is also likely to enhance overall visual health, as suggested by previous study [82]. This is further supported by the Visual Analog Scale (VAS) assessments in our study, which showed a 26.7% improvement in ocular health as perceived by the participants.

Elderberry (*Sambucus* spp.) has traditionally been used to treat respiratory infections, including the common cold and influenza [83,84]. More recently, it has gained attention as a potential preventive measure against COVID-19, as well as for its role in managing post-COVID-19 symptoms [22,23,24]. A systematic review has underscored the potential of elderberry in shortening the duration of these infections, highlighting its relevance in the current pandemic era [85]. The antiviral properties of elderberry are primarily attributed to its high anthocyanin content, a class of flavonoids known for their immunomodulatory and anti-inflammatory properties [86,87]. Anthocyanins are believed to exert their antiviral effects by binding to functional glycoprotein spikes on the viral surface, thereby preventing the virus from entering host cells [31]. Additionally, anthocyanins have also been shown to suppress the Nuclear Factor-κB (NF-κB) pathway, downregulate TLR4 protein expression, and inhibit MAPK signaling pathways, thereby lowering inflammation levels [32]. However, despite these well-documented biological activities, our results indicated that supplementation with elderberry extract at a dose of 100 mg/day for 20 days is not sufficient to provide additional effects in improving the immunity as compared to the placebo group. This observation is in agreement with a few systematic reviews that suggested a minimum dosage of 175 mg/day is required to confer protective effects against infections [85,88,89]. Upon further analysis on the data, it was observed that the changes in ISQ scores in the intervention group (15.9%) were higher than placebo group (10.0%). This suggests that although the dose used in this study was lower than recommended, there may be potential for elderberry extract to improve immunity if administered over a longer duration. This hypothesis is supported by previous research, where anthocyanins showed improved retention in the body after 36 days following a single dose of supplementation [90]. Nonetheless, it is important to note that the additional immune effects observed in the intervention group could also be attributed to the synergistic effects of lutein, zeaxanthin, and elderberry extract. As highlighted in a recent review, the combined consumption of multiple antioxidants often yields greater health benefits compared to consuming individual antioxidants alone [91]. VAS scores displayed a similar pattern, with a 4.44% improvement in self-perceived immunity in the intervention group, with no significant difference from the placebo group. At the present stage, the efficacy of elderberry extract in improving immunity could not be demonstrated in the current study. Further investigations using a higher dosage or longer administration of elderberry extract as a single ingredient are recommended to validate its potential immune-enhancing effects.

## 5. Conclusions

While high doses of lutein and zeaxanthin have demonstrated its protective roles in ocular health, the effects of supplementation at low dosage remain underexplored. This study provides compelling evidence that the combined lutein and zeaxanthin supplementation used can significantly alleviate DED symptoms, with marked improvements in patients’ perceived ocular health. While the effects of supplementation on immunity were not significant at the tested dose and duration, the observed trends suggest potential synergistic benefits from lutein, zeaxanthin, and elderberry extract in enhancing immunity. The findings highlight the critical need for targeted dietary strategies and supplementation to address the burden of DED and related conditions, particularly in populations with insufficient dietary intake of essential nutrients. The use of questionnaires to assess outcomes in this trial may lack objectivity, which should be considered a limitation of the study.

## Figures and Tables

**Table 1:** Characteristics of participants.

Characteristic	Frequency (%)
Gender (*n*/%)	
Male	39 (38.6)
Female	62 (61.4)
Age (years) (*n*/%)	
≤30	14 (13.8)
31–40	21 (20.8)
41–50	48 (47.6)
>50	18 (17.8)
Body Mass Index (BMI) (*n*/%)	
Underweight (<18.5)	7 (6.9)
Normal Weight (18.5–24.9)	51 (50.5)
Overweight (25–29.9)	35 (34.7)
Obese (≥30)	8 (7.9)

**Table 2:** Lutein and zeaxanthin daily intakes by participants.

Food Items	Lutein and Zeaxanthin Concentrations (µg/100 g)	Average Daily Intakes (µg)
Vegetables		
Broccoli (c)	830	23.95
Brussels Sprouts (c)	1290	14.96
Cabbage (r)	310	8.24
Carrots (r)	358	9.41
Collards (c)	8091	56.08
Corn (c)	1800	30.81
Green beans (c)	700	17.13
Green peas (canned)	1350	4.77
Green turnip (c)	8440	39.39
Kale (c)	15,798	140.77
Lettuce(r) (romaine)	2635	74.54
Lettuce (r) (iceberg)	352	6.67
Spinach (c)	7043	110.21
Spinach (r)	11,938	64.12
Squash, winter (c)	66	0.75
Tomatoes (r)	130	3.26
Tomato juice (canned)	60	0.29
Tomato products, puree (canned)	90	0.42
Vegetable juice cocktail	90	0.48
Zucchini (r)	2125	46.29
Fruits ^a^		
Fruit cocktail (canned)	112	2.42
Grapefruit	13	0.07
Melon	40	0.41
Oranges	187	4.47
Orange juice	105	1.10
Papaya	75	0.94
Peach	57	0.27
Tangerine	243	1.99
Tangerine juice	166	0.59
Watermelon	17	0.54
TOTAL		663.49

c: cooked, r: raw. ^a^ All the fruit items have been considered raw.

**Table 3:** Changes in eye health parameters of participant during study; values are expressed as mean ± SD.

Parameters	Group	Pre-Treatment	Post-Treatment	*p*-Value
Ocular Surface Disease Index (OSDI)	Intervention	38.15 ± 11.14	18.26 ± 5.57	<0.05 *
	Placebo	30.54 ± 9.61	22.00 ± 6.60	
Visual Analog Scale (VAS)	Intervention	5.31 ± 1.62	6.73 ± 1.74	<0.05 *
	Placebo	5.72 ± 1.77	6.26 ± 1.61	

Statistically significant *p* values are marked in asterisks (*). *p*-value was calculated using general linear model (GLM) for repeated measures model.

**Table 4:** Changes in immunity parameters of participant during study; values are expressed as mean ± SD.

Parameters	Group	Pre-Treatment	Post-Treatment	*p*-Value
Immune Status Questionnaire (ISQ)	Intervention	7.92 ± 2.45	9.18 ± 1.91	0.495
	Placebo	7.90 ± 3.13	8.69 ± 2.12	
Visual Analog Scale (VAS)	Intervention	6.53 ± 1.59	6.82 ± 1.88	0.622
	Placebo	6.18 ±1.65	6.72 ± 1.72	

*p*-value was calculated using general linear model (GLM) for repeated measures model.

## Data Availability

The datasets generated during and/or analyzed during the current study are available from the corresponding author on reasonable request. All of the individual participant data collected during the trial, after deidentification, will be shared upon reasonable request. Additional documents including study protocol, statistical analysis plan, informed consent form, and clinical study report will also be made available. The data will be available immediately following publication with no end date. Data will be shared with anyone who wishes to access with reasonable request. The data can be used for any type of analyses.
